# COVID‐19 epidemic and mitigation policies: Positive and normative analyses in a neoclassical growth model

**DOI:** 10.1111/jpet.12549

**Published:** 2021-10-21

**Authors:** Luca Gori, Piero Manfredi, Simone Marsiglio, Mauro Sodini

**Affiliations:** ^1^ Department of Law University of Pisa Pisa PI Italy; ^2^ Department of Economics and Management University of Pisa Pisa PI Italy; ^3^ Department of Law University of Naples ‘Federico II’ Naples NA Italy; ^4^ Department of Finance, Faculty of Economics Technical University of Ostrava Ostrava Czech Republic

## Abstract

The COVID‐19 pandemic is still ravaging the planet, but its (short‐, medium‐, and long‐term) diverse effects on health, economy, and society are far from being understood. This article investigates the potential impact of a deadly epidemic and its main nonpharmaceutical control interventions (social distancing vs. testing–tracing–isolation, TTI) on capital accumulation and economic development at different time scales. This is done by integrating an epidemiological susceptible–infectious–recovered model with a Solow‐type growth model including public expenditure, as a parsimonious setting to offer insights on the trade‐off between protecting human lives and the economy and society. The work clarifies (i) the long‐term interactions amongst a deadly infection, demography, and capital accumulation, (ii) the lack of viability of persistent social distancing measures also using an analytical characterization, and the threat of policy‐enhanced COVID‐19 endemicity, (iii) the potentially high return on investments in TTI activities to avoid future lockdowns and related capital disruption. It also quantifies the welfare effects of a range of policies, confirming a counterintuitive role for tax‐funded preventive investments aimed at strengthening TTI as more desirable interventions than generalized lockdowns.

## 1 INTRODUCTION

Since its debut in China in early 2020, the COVID‐19 pandemic is still ravaging the planet with more than 235 million cases and about 4.8 million deaths (on October 4, 2021; WHO, 2021). After the second wave, threatening Europe and the United States since Fall 2020, and the complications due to the appearance of the first highly transmissible variant (i.e., the *English alpha*‐variant, see Fontanet et al., 2021; Volz et al., 2021), the availability of effective vaccines since January 2021 is offering clear exit perspectives from the emergency in industrialized countries (Hodgson et al., 2021). Nonetheless, the continued emergence of highly transmissible new variants, as the *Indian delta*‐variant, spreading fast in the UK and rapidly becoming dominant elsewhere in Europe since spring 2021, the unpredictable degree of protection of current vaccines against future new strains (Mishra et al., 2021), and the still too low vaccination coverage in low‐resource settings (WHO, 2021), are some established factors implying that the coexistence with COVID‐19 is becoming a medium‐/long‐term issue. This, in turn, raises the need to preserve the complex response apparatus that has allowed to control the epidemic so far. Amongst others, this includes a combination of (i) *testing*, contact *tracing*, and early *isolation* of serious symptomatic individuals (TTI), (ii) social distancing measures (up to generalized *lockdowns*), (iii) strengthening hospital's capacity, and (iv) continuing to promote large‐scale information campaigns to maintain public awareness (Ferguson et al., 2020; Walker et al., 2020).

Likewise, it is important to investigate both theoretically and empirically the critical issue that emerged with COVID‐19, namely, the protection of health—from the disease—and of the economy (and of the society as a whole)—from the harmful impacts of the measures enacted for epidemic control (Acemoglu et al., 2020; Alvarez et al., 2021).

During the first pandemic year, the aforementioned measures were applied at widely different intensities amongst world countries and regions (WHO, 2021). About the European experience, national lockdowns aimed at downturning the epidemic course (Walker et al., 2020)^1^ have been the rule during the first wave, with noteworthy exceptions, for example, Sweden that opted for milder social distancing measures jointly encouraging correct individual behavior. The second wave, instead, showed more articulated features mainly due to tier‐based intervention policies (Pradelski & Oliu‐Barton, 2020).

Since the beginning of the pandemic, economists have wondered about the effects of continued large‐scale mitigation measures on gross domestic product (GDP) and employment. Along with epidemic control interventions, expansionary monetary and fiscal policies are being designed to support the economic activity over the entire crisis. Time and research will therefore be necessary to appropriately identify and estimate the direct and indirect effects of the pandemic in the short, medium, and long terms, as basically no sector of social, economic, health, and cultural life can prove unaffected, ranging from work, inequality, social cohesion, mental health, delayed or lost prevention programs, and so on (ILO, 2020; Sumner et al., 2020).

Notwithstanding the explosion of works studying the COVID‐19 pandemic, an “economic epidemiology of infectious disease” was developing since decades having the dynamics of vaccine‐preventable infections and their complications (Philipson, 2000, and references therein) and the dynamics of HIV/AIDS in both developed and developing countries (see the review in Gori et al., 2020, 2021) as main foci. The COVID‐19 pandemic has provided dramatic momentum to the discipline with an endless list of contributions (both theoretical and empirical). The first ones appeared during the earlier phases of the pandemic have combined economic frameworks with epidemiological transmission models (often relying on the classical *susceptible–infectious–recovered*, SIR, model) to study (i) how infection spread might affect individual decisions in goods and labor markets and the ensuing effects of containment policies on employment and GDP (Eichenbaum et al., 2020); (ii) the intensity, duration, and consequences (in terms of economic and human losses) of optimal (first‐best) mitigation measures (Acemoglu et al., 2020; Alvarez et al., 2021; La Torre et al., 2021); (iii) the welfare effects of different combinations of social distancing and testing in a heterogeneous SIR model mimicking the key population age groups (Gollier, 2020).

This article aims at investigating the short‐, medium‐, and long‐term effects of a serious infectious disease initiated in epidemic form but having endemic potential—as COVID‐19—on the pace of capital accumulation and economic development. With this aim, we combine the Solow neoclassical growth model (Solow, 1956) with an epidemiological SIR model endogenously including the main nonpharmaceutical intervention measures adopted against COVID‐19 before the arrival of vaccines. The Solow model was adopted in view of the rapid spread of the SARS‐CoV‐2 virus and of the subsequent emergency measures, which represent negative externalities preventing optimal economic responses by individuals and policymakers. The generalized lockdown enforced by many European governments during the first pandemic wave has forced employees and employers to accept strict measures with little room for behavioral adaptation. Of course, this does not mean that agents’ behaviors were irrelevant (see, e.g., Google mobility data; Google, 2020), indeed their compliance to social distancing possibly contributed to eventually controlling both the first and second pandemic waves. However, individual economic choices were often overwhelmed by centrally planned decisions, generating complicated responses both in consumption and saving (for the Italian case, see ISTAT, 2020).

Our model tries to mimic the main nonpharmaceutical policy responses of Western countries to the pandemic threat, namely, TTI and the social distancing measures (including lockdown), to offer theoretical qualitative insights on both positive and normative aspects of policy interventions against COVID‐19. For the sake of simplicity, the enacted social distancing measures are assumed to be constant over each temporal window of intervention by setting the rate of adequate contacts to a constant level consistent with the desired target of the (current) reproduction number of the infection (${ℛ}_{t}$). The link between the epidemic and the economic subsystems is represented by the effects of social distancing measures on labor and goods markets. These effects are endogenized by combining intervention thresholds based on epidemic severity with balanced budget policies (tax and spending) yielding a trade‐off between epidemic control and economic growth. Through a positive approach, we investigated the effects of different intervention scenarios. Through a normative approach, we quantified the welfare effects of different combinations of interventions to assess their second‐best relative desirability.

The rest of the article proceeds as follows. Section 2 presents the modeling framework. Section 3 studies the model in the absence of feedback effects from the economy to the epidemic. Section 4 considers a positive analysis of mitigation measures. The normative analysis is postponed to Section 5. Concluding remarks follow in Section 6. The Supporting Information in the online appendix reports some mathematical details and extends the normative analysis with additional hypotheses.

## 2 A GENERAL SOLOW‐TYPE ECONOMY WITH AN SIR DISEASE

This section aims at combining the neoclassical growth model (Solow, 1956) with a SIR model aiming to qualitatively capture several features of COVID‐19 transmission and interventions over both the short and the medium/long terms.

### 2.1 The epidemiological model

Population N(t) is divided into three classes S(t),I(t), and R(t), representing the number of susceptible, infected, and recovered (and permanently immune) individuals, respectively, with N(t)=S(t)+I(t)+R(t), where $t\in R_{+}$ denotes time. The population is assumed to obey exponential dynamics in the absence of infection, where b,μ>0 represent the birth rate and the general mortality rate, respectively. We rule out vertical transmission (so far broadly excluded for COVID‐19), so that new‐born enters the class of the susceptible. The population is assumed to mix homogeneously with a per‐capita contact rate M(t), representing the number of distinct individuals encountered per unit of time by each member of the population, so that M(t)I(t) represents the total number of contacts by all infected people at t. Therefore, the total number of *adequate* contacts between infected and susceptible is $M\left(t\right)I\left(t\right)\frac{S\left(t\right)}{N\left(t\right)}$, where $\frac{S\left(t\right)}{N\left(t\right)}$ is the susceptible proportion in the population. If only a fraction p(t) of adequate contacts yields new infections, the overall incidence rate amongst susceptible individuals is $p\left(t\right)M\left(t\right)I\left(t\right)\frac{S\left(t\right)}{N\left(t\right)}=\beta \left(t\right)\frac{I\left(t\right)S\left(t\right)}{N\left(t\right)}$, where β(t)≔p(t)M(t)>0 is the transmission rate. *The time dependency in the transmission rate* is assumed to reflect the effects of social distancing due to both public interventions and individuals' spontaneous behavioral changes in response to the epidemic threat. Infected individuals are assumed to recover and acquire immunity at a constant rate γ>0, and to suffer the excess mortality induced by the infection at rate $\mu_{c}>0$. Testing, tracing, and isolation after diagnosis remove the infected at an effective endogenous rate θ(G(t),I(t)), where G(t)>0 is the level of extraordinary public expenditure specifically targeted for COVID‐19 testing, tracing, and isolation. The dependency on the infective population aims at primarily reflecting the finiteness of testing equipment and personnel. This amounts to assuming that all tested individuals recover to reflect that testing activities mostly identify asymptomatic individuals.^2^

The general epidemiological model reads as follows:

(1) $$\left\{\begin{array}{l} \dot{S}\left(t\right)=bN\left(t\right)-\mu S\left(t\right)-\beta \left(t\right)\frac{I\left(t\right)S\left(t\right)}{N\left(t\right)},\\ \dot{I}\left(t\right)=\beta \left(t\right)\frac{I\left(t\right)S\left(t\right)}{N\left(t\right)}-\left[\gamma +\mu +\mu_{c}+\theta \left(G\left(t\right),I\left(t\right)\right)\right]I\left(t\right),\\ \dot{R}\left(t\right)=\left[\gamma +\theta \left(G\left(t\right),I\left(t\right)\right)\right]I\left(t\right)-\mu R\left(t\right), \end{array}\right.$$

where the dots denote time derivatives. Total population N(t) evolves over time according to the equation:

(2) $$\dot{N}\left(t\right)=\left(b-\mu \right)N\left(t\right)-\mu_{c}I\left(t\right).$$

To sum up, the model includes: (i) a nontrivial vital dynamics of the population (by taking b≠μ) to consider the potential effects of COVID‐19 and related interventions beyond the short term; (ii) COVID‐specific mortality; (iii) testing activities aimed at confirming and isolating active COVID‐19 cases. This allows us to capture the two main currently deployed public interventions (testing and social distancing), and the long‐term effects of the interplay between infection transmission, demography, and policy interventions. The dependence of the testing rate on public spending is a critical idea especially concerning the medium term, as the possibility to contain future epidemic reemergence avoiding the resort to new lockdown policies will strategically depend on the capability of the public system to improve “local” prevention by promoting highly effective TTI. Notably, the model also captures very short‐term effects by setting b=μ=0.

Remark 1 The model in (1) represents the epidemiology of COVID‐19 in a simplified way. First, it postulates acquisition of permanent immunity, which is not necessarily the case though evidence in this sense is still missing. Second, it disregards both the dimensions related to the treatment of COVID‐19 (which would require including at least hospitalizations) and age‐heterogeneities, by which the onset of symptoms and the development of serious COVID‐related illnesses are sharply increasing with age. Then, this toy model aims at mimicking COVID‐19 trends only in the adult/working population, which is known to have a high infection probability in the absence of control measures but a rather small probability of serious consequences and mortality.

### 2.2 A general Solow‐type model

Let us now move to the narrative of the economic side of the article by directly following Solow (1956). There exists a one‐sector closed economy producing a homogeneous good Y(t) that can be used for consumption and (gross) investment purposes. Everything that is not consumed is therefore invested to build on new units of physical capital K(t) that depreciates at rate δ>0. Production at time t of the homogeneous good Y(t) takes place according to the standard constant‐return‐to‐scale Cobb–Douglas technology combining capital and labor according to $Y\left(t\right)=F\left(K\left(t\right),L\left(t\right)\right)=AK{\left(t\right)}^{\alpha }L{\left(t\right)}^{1-\alpha }$, where capital K(t) represents the durable physical inputs and L(t) the labor force employed at the aggregate level, 0<α<1 is the output elasticity of capital and A>0 is a parameter that scales up/down technology and represents a measure of technological progress. The net increase in the stock of physical capital at every moment in time is given by gross investment minus the portion of capital that cannot be re(used) because of depreciation (δK(t)). As the equilibrium condition is given by gross investment equals aggregate saving (X(t)), the basic dynamics of capital is described by

(3) $$\dot{K}\left(t\right)=xY\left(t\right)-\delta K\left(t\right)=xAK{\left(t\right)}^{\alpha }L{\left(t\right)}^{1-\alpha }-\delta K\left(t\right),$$

where 0<x<1 is the constant marginal propensity to save (or the fraction of income invested in capital accumulation).

To account for public intervention against COVID‐19, we assume that COVID‐specific public expenditure G(t) finances TTI activities at a balanced budget using labor and capital income taxes. Therefore, government expenditure G(t) equals tax revenues T(t)=τY(t), where 0≤τ<1 is the constant tax rate. Therefore, the budget constraint is given by

(4) G(t)=g(t)N(t)=τY(t),

where g(t) is the per‐capita public expenditure. Combining the dynamics of capital (3) with the government budgets (4), one gets

(5) $$\dot{K}\left(t\right)=x\left(1-\tau \right)Y\left(t\right)-\delta K\left(t\right)=x\left(1-\tau \right)AK{\left(t\right)}^{\alpha }L{\left(t\right)}^{1-\alpha }-\delta K\left(t\right).$$

### 2.3 Integrating the economic and epidemiological systems

The presence of the SARS‐CoV‐2 virus and the existence of related control measures affect the labor force in a twofold way. First, all the infected are assumed to be isolated (quarantined) and unable to work. Second, lockdown measures prevent a fraction of healthy workers from going to work. Define now 0<q(t)<1 as the share of “fundamental” workers mandatorily compelled to work. Therefore, the size of the labor input definitively employed in production is L(t)=q(t)[S(t)+R(t)]=q(t)[N(t)−I(t)] and the dynamics of the capital stock is given by

(6) $$\dot{K}\left(t\right)=x\left(1-\tau \right)AK{\left(t\right)}^{\alpha }{\left\{q\left(t\right)\left[N\left(t\right)-I\left(t\right)\right]\right\}}^{1-\alpha }-\delta K\left(t\right).$$

The full model is obtained by combining Equations (1) and (6). Parameter q(t) is assumed to directly reflect social distancing interventions aimed at reducing transmission based on a homogeneous mixing hypothesis: q(t)=ζβ(t), where ζ is a positive constant (see Section 4.1).

## 3 ECONOMIC CONSEQUENCES OF AN UNCONTROLLED INFECTIOUS DISEASE: NO FEEDBACK EFFECTS OF THE ECONOMY ON THE INFECTION

This section investigates the long‐term effects of an uncontrolled epidemic on capital accumulation in the basic Solow model. This is obtained by setting τ=0 in Equation (6), θ(G(t),I(t))=0 in (1) and assuming that all healthy workers (N(t)−I(t)) are employed (q(t)=1 in Equation 6). In this case, the epidemiological system affects the economy but not vice versa. This allows us to derive analytical predictions about the effects of a deadly infection on economic outcomes.

### 3.1 Theoretical results

Under the above assumptions, the epidemiological component (1) collapses into the following classical model for endemic infections with a variable population (Busenberg & van den Driessche, 1990):

(7) $$\left\{\begin{array}{l} \dot{S}=bN-\mu S-\beta \frac{IS}{N},\\ \dot{I}=\beta \frac{IS}{N}-\left(\gamma +\mu +\mu_{c}\right)I,\\ \dot{R}=\gamma I-\mu R, \end{array}\right.$$

plus the population equation (2). As the population is nonstationary, let us introduce the fractionary variables s=S∕N,i=I∕N,r=R∕N (so that s+i+r=1) and the per‐capita stock of capital k=K∕N. The models (1) and (6) can be rewritten as

(8) $$\left\{\begin{array}{l} \dot{s}=b+\left[\left(\mu_{c}-\beta \right)i-b\right]s,\\ \dot{i}=i(\beta s+\mu_{c}i-b-\gamma -\mu_{c}),\\ \dot{N}=\left(b-\mu \right)N-\mu_{c}I,\\ \dot{k}=xA\left(1-\tau \right)k^{\alpha }{\left[q\left(1-i\right)\right]}^{1-\alpha }-\left(b-\mu -i\mu_{c}+\delta \right)k. \end{array}\right.$$

As the equilibria of the *si* epidemiological equations in (8) are globally stable, the system (8) is asymptotically autonomous in the sense of Thieme (1992). Therefore, its long‐term dynamics can be characterized by noting that the epidemiological component will tend to a unique globally stable steady state and subsequently analyzing the economic component by plugging the infective equilibrium into Solow's equation.

The epidemiological equations in (8) converge either to the infection‐free equilibrium $E_{0}=\left(1,0,0\right)$ or to the unique endemic equilibrium $E_{1}=\left(s^{*},i^{*},r^{*}\right)$ and its global dynamics depend on the following thresholds (Busenberg & van den Driessche, 1990):

(9) $${ℛ}_{0}=\frac{\beta }{b+\gamma +\mu_{c}},$$

(10) $${ℛ}_{1}=\left\{\begin{array}{ll} \frac{b}{\mu } & \;\text{if}\;\;{ℛ}_{0}\leq 1,\\ \frac{b}{\mu +\mu_{c}i^{*}} & \;\text{if}\;\;{ℛ}_{0}>1, \end{array}\right.$$

and

(11) $${ℛ}_{2}=\left\{\begin{array}{ll} \frac{\beta }{\mu +\mu_{c}+\gamma } & \;\text{if}\;\;{ℛ}_{0}\leq 1,\\ \frac{\beta s^{*}}{\mu +\mu_{c}+\gamma } & \;\text{if}\;\;{ℛ}_{0}>1. \end{array}\right.$$

The reproduction number ${ℛ}_{0}$ governs whether the infective prevalence dies out or becomes endemic, whereas ${ℛ}_{1}$ and ${ℛ}_{2}$ are further thresholds appearing because of the interplay between the epidemiological and demographic evolutions (see Busenberg & van den Driessche, 1990, for further details). The main results of the epidemiological subsystem in (8) are as follows. The infection‐free equilibrium exists for all parameter values, while the endemic equilibrium exists only if ${ℛ}_{0}>1$. The infection‐free equilibrium is globally asymptotically stable when it is unique (i.e., ${ℛ}_{0}\leq 1$). The infection‐free equilibrium becomes unstable and the endemic state inherits the global stability properties when also the endemic equilibrium exists (i.e., ${ℛ}_{0}>1$). The condition ${ℛ}_{0}=1$ represents a transcritical bifurcation at which the number of equilibria along with their stability properties change. This means that according to the size of ${ℛ}_{0}$ the infection might tend to die out or persist into the population in fractionary terms. Therefore, understanding its determinants is critical for the subsequent policy analysis. Specifically, the reproduction number ${ℛ}_{0}$ increases with the transmission rate (β) and decreases with the recovery rate (γ), the birth rate (b), and the infection‐specific mortality rate ($\mu_{c}$). In the event of an epidemic outbreak, policy actions affecting the numerator and the denominator of ${ℛ}_{0}$ can be used to reduce it below unity to ensure complete eradication over the long term.

The analysis of the overall system (8) shows that there can be at most two equilibria, namely, an equilibrium where the economy is infection‐free

(12) $$\left(s,i,r,k\right)=\left(1,0,0,\tilde{k}\right),$$

and an equilibrium where the infection establishes endemically in the economy

(13) $$\left(s,i,r,k\right)=\left(s^{*},i^{*},r^{*},k^{*}\right).$$

In particular, $i^{*}$ is the unique positive real root (when it exists) of the following polynomial:

(14) $$P\left(i\right)≔\Gamma i^{2}+\Delta i+\Theta ,$$

with $\Gamma ≔-\mu_{c}\left(\beta -\mu_{c}\right),\Delta ≔\left(\beta -\mu_{c}\right)\left(\mu_{c}+\gamma +b\right)-b\mu_{c}$, and $\Theta ≔b\left(b-\beta +\mu_{c}+\gamma \right)$. In addition, we have that

(15) $$s^{*}=\frac{b}{\beta i^{*}-i^{*}\mu_{c}+b},$$

(16) $$r^{*}=\frac{\gamma }{\beta -i^{*}\mu_{c}},$$

(17) $$k^{*}=\left(1-i^{*}\right){\left[\frac{-i^{*}\mu_{c}+b+\delta -\mu }{Ax}\right]}^{\frac{1}{\alpha -1}},$$

(18) $$\tilde{k}={\left[\frac{b+\delta -\mu }{Ax}\right]}^{\frac{1}{\alpha -1}}.$$

By considering the expressions just introduced in (13) and (12), the following proposition holds.

Proposition 2 Consider the epidemiological Solow model described by the expressions in (8). Then, we have that:

(a) The infection‐free equilibrium of the economy $\left(s,i,r,k\right)=\left(1,0,0,\tilde{k}\right)$ always exists. It is unique and globally asymptotically stable in the feasible region
(19) $$ℱ≔\left\{\left(s,i,r,k\right)\in {ℜ}^{4}:s\geq 0,i\geq 0,r\geq 0,s+i+r=1,k>0\right\},$$
  for ${ℛ}_{0}\leq 1$. It is unstable for ${ℛ}_{0}>1$.
(b) When ${ℛ}_{0}>1$, there exists a unique endemic equilibrium of the economy $\left(s,i,r,k\right)=\left(s^{*},i^{*},r^{*},k^{*}\right)$ with $i^{*}>0,r^{*}>0$, and $k^{*}>0$, which is globally asymptotically stable in region ℱ.
(c) The asymptotic behavior of the total population N(t) is N(t)→0 if ${ℛ}_{1}<1$, and N(t)→∞ if ${ℛ}_{1}>1$.
(d) When ${ℛ}_{1}>1$, the asymptotic behavior of the total infected population I(t) is I(t)→0 if ${ℛ}_{2}<1$, and I(t)→∞ if ${ℛ}_{2}>1$.

The proof is in the online appendix.  □

The relationship between epidemiological and economic variables in (8) is not straightforward. The infection prevalence i affects the dynamics of k by generating two opposing effects. On the one hand, an increase in prevalence directly reduces both the workforce and production. On the other hand, due to the disease‐specific mortality $\mu_{c}$, an increase in i reduces the population growth rate (i.e., $b-\mu -i\mu_{c}$), causing in turn an increase in k. This is because the higher mortality rate reduces the population growth rate so that the capital stock K is distributed over a lower number of individuals (capital‐dilution effect). The net effect of these factors depends on the characteristic of the infection. In plain words, the interplay between transmission and accumulation passes through two counterbalancing components: (a) the reduction in labor force participation caused by the forced quarantine of the infected, which directly reduces production, capital accumulation, and the stock of capital per capita (this process displays constant returns), and (b) the reduction in the population growth rate that COVID‐19 mortality may generate, which in turn increases the stock of capital per capita by increasing the wage rate and sustaining indirectly capital accumulation (this process displays decreasing returns). The outcome definitively depends on which of the two forces dominates though the latter one is generally weaker than the former. If the indirect effect is strong enough to offset the reduction in the workforce brought about by premature death, an increase in infections increases capital accumulation.

### 3.2 A calibration on Italian COVID‐19 data

We now illustrate the previous analysis by calibrating the model to the Italian economy and its COVID‐19 experience (parameter values are summarized in Table 1). As for economic parameters, the depreciation rate of capital (δ) was set to 2%/year, the propensity to save (x) to 10% (Jappelli et al., 2014), the output elasticity of capital to α=0.3 (Gollin, 2002), and the total factor productivity to A=30. The rate of change of the labor supply in the absence of infection was set to n≔b−μ (the population growth rate) under the assumption b=μ, describing a stationary population. As for epidemiological parameters, we set ${ℛ}_{0}≅3$ as approximately observed in several Italian regions during the first wave (Riccardo et al., 2020) and derive the transmission rate of a free epidemic β from (9). The recovery rate γ was set in the range of 5–7 days (Riccardo et al., 2020; Walker et al., 2020). Finally, the infection specific mortality of adult workers ($\mu_{c}$) was chosen (given γ) by assigning a 99% conditional probability that an adult recovers from COVID‐19 infection ($\gamma ∕\left(\gamma +\mu_{c}\right)=0.99$). The resulting low value of $\mu_{c}$ is consistent with the low COVID‐19 mortality amongst the overall working population. Under b=μ previous assignments imply a slowly decaying population in the long‐term endemic regime.

**Table 1:** Parameter values employed in the numerical simulations

Parameter	Unit	Baseline value or range	Source
A	Adim.	30	Arbitrary (pure scaling parameter)
α	Adim.	0.3	Krueger (1999) and Gollin (2002)
δ	${\mathrm{day}}^{-1}$	0.02∕365	Literature
x	Adim.	0.1∕365	Literature
${ℛ}_{0}$	Adim.	≅3.0	Italy 2020 (Nat. Inst. of Health)
β	${\mathrm{day}}^{-1}$	3γ	Assigned from (9)
μ	${\mathrm{day}}^{-1}$	1∕(80⋅365)	Average life expectancy (Italy, 2019)
b	${\mathrm{day}}^{-1}$	μ	See main text
$\mu_{c}$	${\mathrm{day}}^{-1}$	0.001683	Chosen as a simulation parameter
γ	${\mathrm{day}}^{-1}$	1∕6	Early Chinese studies

*Note*: Adim., nondimensional parameter. All per‐capita rates are reported on the common unit of day^−1^.

**Figure 1:**
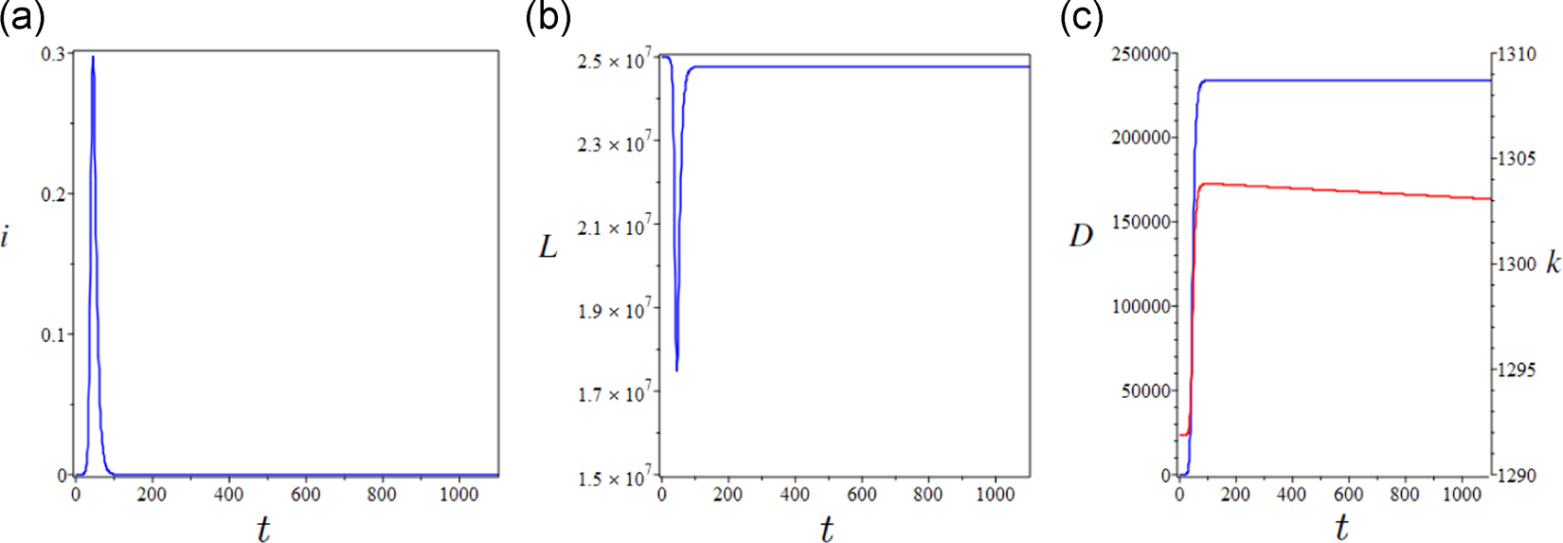
Short‐term epidemiological and economic effects of an uncontrolled epidemic (over a 3‐year horizon). (a) Infection prevalence (i), (b) the drop in labor demand, and (c) cumulative COVID‐specific mortality (blue curve) and capital accumulation trend (red curve). Initial conditions: $i_{0}=15∕N_{0},r_{0}=0,N_{0}=\text{25,000,000}$. Time is measured in days

The epidemic was initialized in a fully susceptible working population at full employment (L=N) at its long‐term Solovian equilibrium of capital per capita ($k^{*}=1284$). Given full initial susceptibility, the impact of the first epidemic wave (Figure 1a) yields a sharp short‐term drop in the working population (Figure 1b) and a large absolute number of deaths. However, the drop in production is mild due to the short duration of the epidemic and its effect is offset by the mortality bulge. This temporarily pushes upward capital accumulation (Figure 1c). Overall, the quantitative impact on capital accumulation is small due to the low relative impact of adult mortality.

**Figure 2:**
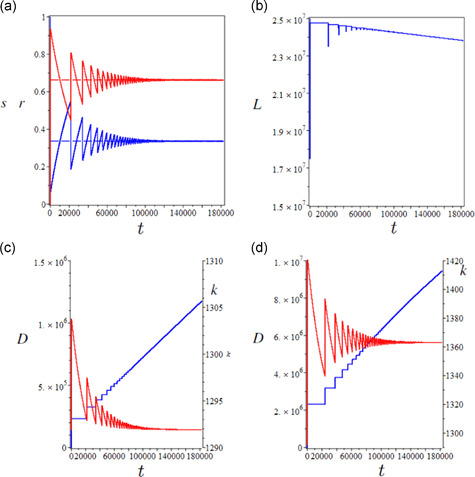
Long‐term epidemiological and economic effects of an uncontrolled epidemic. (a) Susceptible (blue curve) and recovered (red curve) proportions, (b) effective labor, (c) cumulative COVID‐19 deaths (blue curve) and capital accumulation (red curve), and (d) as (c) but with a 10‐fold higher infection‐specific mortality, eventually bringing a sharp positive effect on accumulation. Time is measured in days

Consistent with our theoretical predictions, the infection will eventually become endemic in the long term with the susceptible and immune proportions converging (Figure 2a) towards their steady‐state values (15) and (16). This occurs through a sequence of damped epidemic waves, as well known in basic epidemiological theory. Convergence towards the equilibrium requires over 200 years. The recurring epidemics continue to cause (damped) short‐term shocks in effective labor (Figure 2b). The population will asymptotically decline due to the COVID‐specific mortality, but this effect is mild due to the assumed low level of $\mu_{c}$. The resulting long‐term dynamics of capital accumulation are essentially unaffected (Figure 2c). A counterfactual experiment with a 10‐fold higher infection‐specific mortality would unexpectedly lead to a much larger increase of per‐capita capital accumulation (Figure 2d). This beneficial effect of the epidemic on capital accumulation was termed the “world of opportunities after the Black Death” by Young (2005; see also Gori et al., 2020) for HIV/AIDS in South Africa.

## 4 PUBLIC INTERVENTION PROGRAMS: FEEDBACKS OF THE ECONOMY ON THE INFECTION

This section considers public interventions against a deadly infection and the ensuing feedbacks from a *positive* viewpoint. In particular, it analyzes separately the two main types of interventions adopted against COVID‐19 before the arrival of vaccination, namely, social distancing and TTI. The TTI is entirely financed via an ad hoc income taxation, while there are no direct costs of social distancing, which is modulated through reductions in the transmission rate β(t). Our baseline scenario considers an exogenous intervention on social contacts for (abruptly) achieving a desired *constant* level of the *lockdown reproduction number* $R_{L}$. We set $R_{L}=0.6$, which is not distant from the values achieved during the 2020 national lockdown in many Italian regions when social distancing entered its regime phase (Riccardo et al., 2020). Using the relationship q(t)=ζβ(t), we assume that the reduction in transmission (−80%) is reflected into a corresponding reduction in the workforce ($q\left(t\right)=q_{L}=0.2$). This is consistent with the hypothesis of homogeneous mixing by which humans meet each other at the same rate regardless of the activity considered (work, school, leisure, etc.). The lockdown is declared during the phase of exponential epidemic increase when a *locking threshold* ($T_{L}$) defined on infection prevalence is achieved. We set $T_{L}=1∕15,000$ corresponding to a prevalence of 6.5 per 100,000 or an infection incidence of about 1 per 100,000 per day. This hypothesis follows early COVID‐19 studies (Ferguson et al., 2020), where the approach to locking/reopening was only based on incidence. Subsequent studies suggested the need for more involved approaches, as the “color” system adopted after the end of the first wave. Pairwise, the *unlocking threshold* ($T_{U}$) is set either to $T_{U}^{\mathrm{Low}}=1∕\text{200,000}$ or $T_{U}^{\mathrm{High}}=1∕20,000$ to mirror two distinct attitudes to reopening, namely, a prudential attitude requiring a substantial prevalence decline before unlocking ($T_{U}^{\mathrm{Low}}$) versus an opposite one, more biased towards protecting from economic/societal impacts of lockdown ($T_{U}^{\mathrm{High}}$). Previous hypotheses imply that the current reproduction number and the fraction q(t) during the *lockdown window* $\left(T_{L},T_{U}\right)$ are set to the constants $R_{L}$ and $q_{L}$, respectively, while they are restored to their *natural* values ${ℛ}_{0}$ and q=1 after intervention relaxing. These parameter assignments are summarized in Table 2.

**Table 2:** Parameter values employed in the numerical simulations

Parameter	Unit	Baseline value or range	Source
τ	Adim.	0	Free simulation parameter
q	Adim.	[0.2,1]	Proportional to the factor ${ℛ}_{L}∕{ℛ}_{0}$
$R_{L}$	Adim.	0.6	Italy 2020 (Nat. Inst. of Health)
$T_{L}$	Adim.	1∕15,000	See main text
$T_{U}^{\mathrm{High}},T_{U}^{\mathrm{Low}}$	Adim.	1∕20,000,1∕200,000	See main text

*Note*: Adim., nondimensional parameter. All per‐capita rates are reported on the common unit of day^−1^.

### 4.1 Pure lockdown policies and capital accumulation: A baseline scenario

**Figure 3:**
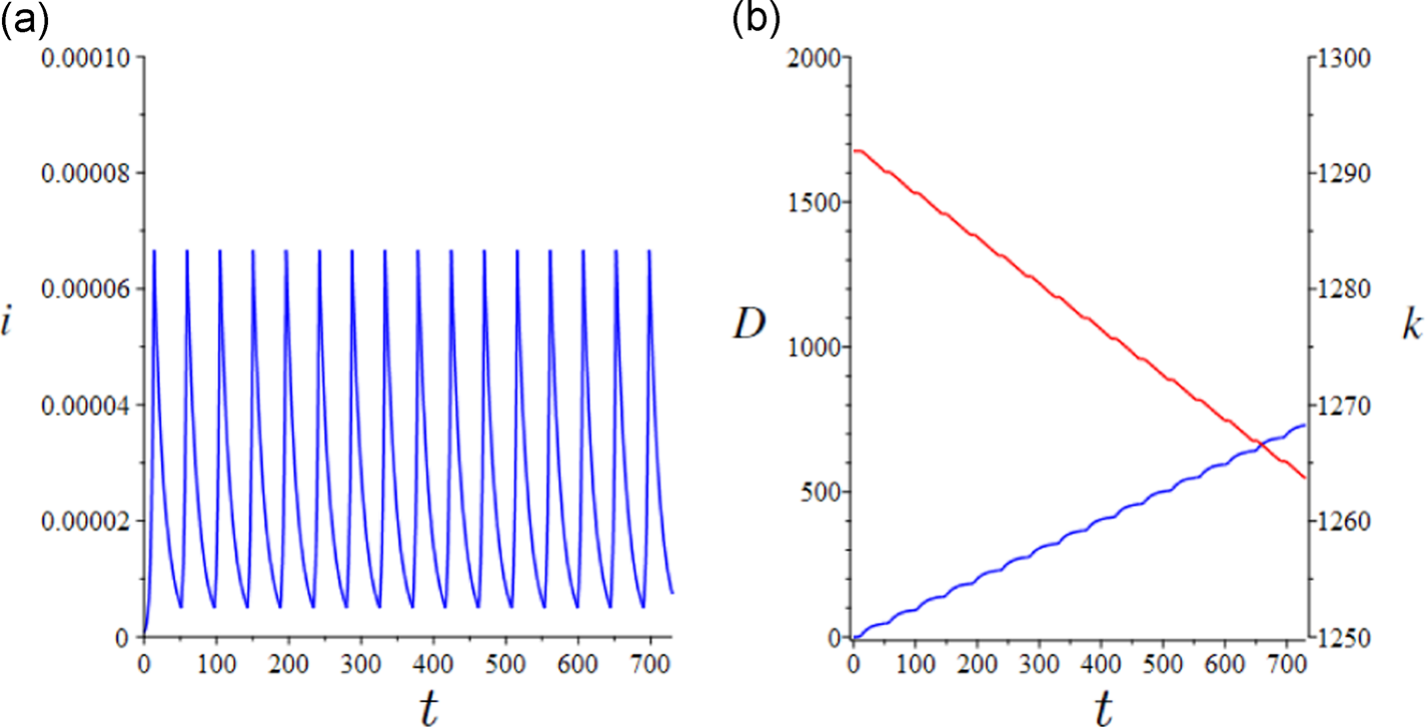
The pure lockdown baseline scenario: Short‐term epidemiological and economic effects in the prudential option ($T_{U}=T_{U}^{\mathrm{Low}}$). (a) Lockdown waves of infection prevalence over a 2‐year period and (b) cumulative deaths (blue curve), $D=\mu_{c}I$, and capital accumulation (red curve). Time is measured in days

**Figure 4:**
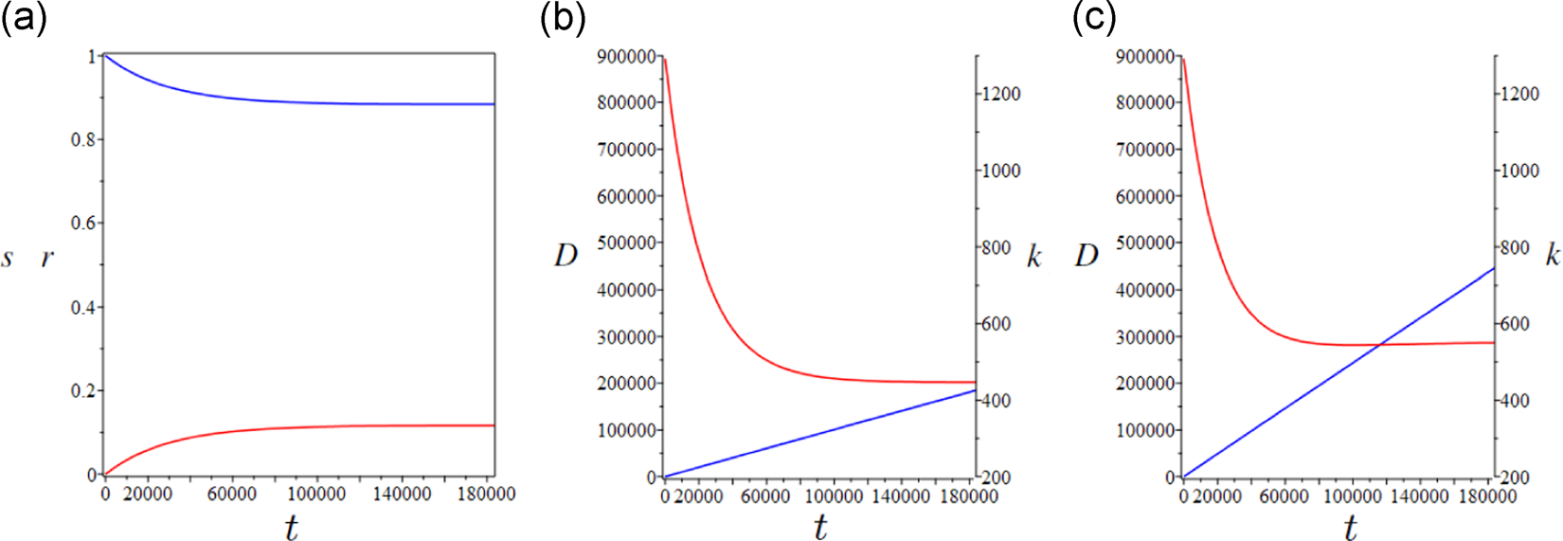
The pure lockdown baseline scenario: Long‐term epidemiological and economic effects. (a) Susceptible (blue curve) and recovered (red curve) proportions, (b) cumulative deaths (blue curve), $D=\mu_{c}I$, and capital accumulation (red curve) in the prudential option ($T_{U}=T_{U}^{\mathrm{Low}}$), and (c) as (b) with $T_{U}=T_{U}^{\mathrm{High}}$. Time is measured in days

The epidemic is initialized from the same conditions as the free uncontrolled scenario. By focusing on the prudential option ($T_{U}=T_{U}^{\mathrm{Low}}$), this *pure lockdown* policy (i.e., in the absence of TTI) yields a sequence of “lockdown induced waves” of infection prevalence (Figure 3a) first described for COVID‐19 by Walker et al. (2020), with several lockdown cycles throughout the year. Each lockdown cycle brings a dramatic positive effect in terms of avoided deaths on the one hand, and a decline in capital accumulation due to the loss in production on the other hand (Figure 3b). In this case, the fall in production dominates the capital widening effect observed for the free epidemic scenario. Over the long term, the pure lockdown policy artificially sustains endemicity with the susceptible and immune proportions setting to levels (Figure 4a) far from those typical of an endemic infection (Figure 1a). The harmful effects on accumulation cumulate over the years yielding a long‐term decline in capital per capita (Figure 4b). The short‐(and medium‐/long‐)term impact of a sustained lockdown policy is modulated by the relative duration of the lockdown period, as clear by a comparison—other things being equal—between the scenarios $T_{U}=T_{U}^{\mathrm{Low}}$ and $T_{U}=T_{U}^{\mathrm{High}}$ (Figure 4c). The lower decline in accumulation trades‐off with a higher mortality burden.

### 4.2 Lockdown and accumulation at low epidemic attack rates: Closed‐form results

In view of its tractability, the Solow model allows one to make—under suitable hypotheses—simple analytical predictions on the economic consequences of a *recurrent* social distancing policy (as those depicted in Figure 3) on capital accumulation. In particular, we will consider here the case of successful social distancing allowing to keep epidemic attack rates at low levels, so that the susceptible proportion never deviates significantly from 100%. In this case, by setting s=1 in the epidemiological equations, the dynamics of infectives during a single lockdown/unlocking phase are exponential (increasing and declining, respectively, as depicted in Figure 3a), and the dynamics over a sequence of such phases is essentially periodic due to the fixed locking and unlocking thresholds $\left(T_{L},T_{U}\right)$. Under this hypothesis, the infection impact on effective labor and the mortality impact on accumulation are negligible, and all the effects on the economy are due to the fall in production during the lockdown phases. For the sake of simplicity, let us assume that the dynamics are exactly periodic, with locking/unlocking phases of fixed duration Q, of which a proportion χ is spent in lockdown, therefore at a reduced proportion $q=q_{L}$ of the labor force employed, while the rest of the period, having duration (1−χ)Q, is a regular production period (q=1). Previous considerations, and the approximation 1−i≈1 (following from the postulated strong containment of the epidemics) imply that the relevant equation of the per‐capita stock of capital (8) can be approximated as follows:

(20) $$\dot{k}=x\left(t\right)Ak^{\alpha }-\left(n+\delta \right)k,$$

where function x(t) denotes the effective (time‐dependent) savings rate resulting during the lockdown cycles: $x\left(t\right)=x\cdot {\left(q\left(t\right)\right)}^{1-\alpha },n≔b-\mu$, and $q\left(t\right)=q_{L}$ during lockdown periods while q(t)=1 during normal production periods. This implies x(t)=x for normal production periods, and $x\left(t\right)=x_{L}$ for lockdown periods. Briefly, the resulting Solow‐type model therefore has periodic, piecewise‐constant, effective saving, which takes on its ordinary value x during “normal” phases, and the reduced value $x_{L}$ during lockdown phases. Equation (20) is a Bernoulli equation that can be solved in a closed form like the standard Solow equation, by passing through the equation of capital per unit of output. This is obtained by the usual transformation $z=k^{1-\alpha }$, yielding the equation:

(21) $$\dot{z}=\left(1-\alpha \right)Ax\left(t\right)-\left(1-\alpha \right)\left(n+\delta \right)z.$$

Equation (21) is linear and can be integrated over the subsequent cycles of locking/unlocking starting from whatever initial condition. However, a convenient initial condition is obtained by assuming that before the pandemic outbreak and related mitigation measures the capital–output ratio $z_{0}$ was at its equilibrium level $\hat{z}$ of the model at full employment (q=1) and therefore full‐savings:

(22) $$\hat{z}=\frac{x}{n+\delta }.$$

By repeated integrations over the locking/unlocking cycles of fixed duration Q, one can then evaluate the cumulative loss of the capital–output ratio. After the first cycle it holds

(23) $$z\left(Q\right)=\hat{z}-\Lambda ,$$

where Λ denotes the loss of the capital–output ratio during the first locking/unlocking cycle:

(24) $$\Lambda =\frac{1-\alpha }{n+\delta }\left(1-e^{-\left(n+\delta \right)\chi Q}\right)\left(x-x_{L}\right),$$

which is an increasing function of the time χQ spent in lockdown, and of the difference $\left(x-x_{L}\right)$. After h locking/unlocking cycles, the capital–output ratio will suffer the cumulative loss:

(25) $$z\left(hQ\right)=\hat{z}-\Lambda \frac{\left(1-{\left(e^{-\left(n+\delta \right)\chi Q}\right)}^{h}\right)}{\left(1-e^{-\left(n+\delta \right)\chi Q}\right)}.$$

This nice formula can be used to evaluate in a fully analytical manner the output loss resulting from any number of lockdown cycles under the specific hypotheses made here. Moreover, it can be easily translated into the corresponding loss in per‐capita capital.

### 4.3 Pure TTI policies and lockdown avoidance

Previous results illustrated the trade‐off between COVID‐19 deaths and the protection of the economy that has been a major concern of policymakers and early economic studies on the subject. Here we focus on publicly funded pure TTI programs aimed at preventing large‐scale outbreaks and the subsequent need to lockdown. The underlying idea is to strengthening local public health resources to test, detect, trace, and isolate as many asymptomatic cases as possible to contain any possible outbreak at the onset. This is represented in (1) by the testing function θ(G,I) such that the total confirmation of cases per unit of time θ(G,I)⋅I is: (i) a saturating function of prevalence to reflect the finiteness of diagnostic tools and staff in the very short term, and (ii) an increasing function of public expenditure to reflect the capability of the public health system to expand such resources in the longer term. Here, we make the following choice in per‐capita terms:

(26) $$\theta \left(g,i\right)=\frac{1}{H+i}\cdot \frac{2\gamma g}{B+\eta g},$$

where g=τ⋅f[k,(1−i)q],f is the intensive form production function, the term $\frac{2\gamma g}{B+\eta g}$ is the upper bound of testing, the term $\frac{1}{H+i}$ is decreasing in i and reflects the finiteness of diagnostic tools for increasing prevalence, η>0 tunes the efficiency of TTI and H,B>0 represent scaling parameters.

**Figure 5:**
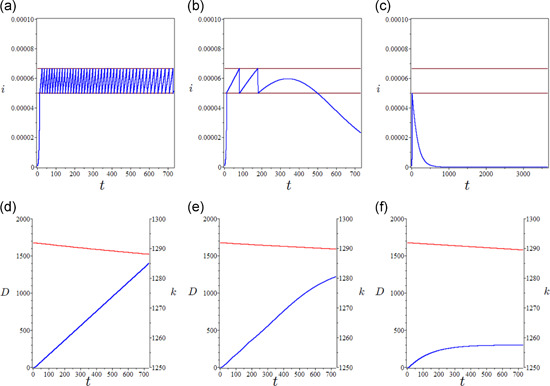
The TTI scenario funded by public expenditure: Short‐term (2 years) epidemiological and economic effects when $T_{U}=T_{U}^{\mathrm{High}}$. (Left panels) τ=0.03, (central panels) τ=0.038, (right panels) τ=0.044, (upper graphs) infection prevalence, and (lower graphs) capital per capita (red curve) and COVID‐19 cumulative excess mortality (blue curve), $D=\mu_{c}I$, and (parameter values) η=1,H=0.78, and B=1. Time is measured in days

The ensuing results are reported in Figure 5 for three qualitatively different outcomes of TTI measures depending on different levels of the targeted public expenditure over a time horizon of 2 years. At low levels of expenditure (τ=0.03), the TTI policy results unable to contain the outbreaks and this calls for continued lockdowns (Figure 5a). At intermediate levels (τ=0.038), TTI is unable to contain the first few epidemic waves but is effective in preventing any further wave and related lockdowns after a certain time point (Figure 5b). Finally, at higher levels of expenditures (τ=0.044), TTI can avoid any outbreak and subsequent lockdown (Figure 5c). This is because the testing rate can bring the reproduction number below the threshold. The corresponding effects on mortality and the per‐capita stock of capital are reported in the lower panels of Figure 5.

## 5 WELFARE EFFECTS

This section assesses the welfare effects of social distancing and TTI measures (either separate or combined) from a normative viewpoint. It includes an appropriate measure of the disutility of COVID‐related mortality within standard formulations of social welfare by considering the integral of the discounted product between the individual instantaneous utility function U(c,d) and a suitable function of the population size, where $c=\frac{C}{N}$ is per‐capita consumption, $d=\frac{D}{N^{\phi }}$ is a mortality indicator where ϕ={0,1}, and $D=\mu_{c}I$ represents the number of COVID‐19 deaths (Hall & Jones, 2007; Hall et al., 2020). The case ϕ=1 considers the COVID‐19 per‐capita mortality rate, whereas ϕ=0 accounts for the overall (nonnormalized) number of deaths. The former case reflects a social planner accounting for per‐capita mortality, suggesting that the disease death represents a private bad. The latter one might also be relevant once an unexpected cause of mortality suddenly occurs, so that even a small number of deaths might yield widespread societal anxiety, as it already happened, for example, with the SARS‐CoV‐1 virus (Person et al., 2004). In this case, the disease‐induced mortality can be interpreted as a public bad. The utility function U is assumed to be increasing and concave in per‐capita consumption, decreasing and convex in the mortality indicator, and additively separable in its two arguments, that is,

(27) U(c,d)=u(c)−ω⋅v(d),

where ω>0 captures the relative weight of the disutility of COVID‐19 mortality (compared with the utility of consumption). The social welfare function reads as follows:

(28) $$W=\int_{0}^{T}\left[u\left(c\right)-\omega \cdot v\left(d\right)\right]N^{\varepsilon }e^{-\rho t}\;dt,$$

where ρ>0 is the rate of time preference and ε={0,1} determines the welfare criterion, that is, total or Benthamite utilitarianism (ε=1) and average or Millian utilitarianism (ε=0). In the Benthamite (resp., Millian) case, social welfare coincides with the sum of all agents' utilities (resp., the representative agent's utility). The implications of these alternative approaches for economic growth outcomes have been discussed by, for example, Canton and Meijdam (1997). Though massive infectious disease‐related deaths can have important welfare implications, especially in low‐resource settings (Gori et al., 2020, 2021), the currently observed COVID‐19 mortality burden will have minor effects on the overall population meaning that its remarkable aggregate welfare effects will not be significantly affected by the adopted utilitarian criterion (as will become clear in the subsequent sections).

Public policies aiming at reducing the spread of SARS‐CoV‐2 infection, as lockdown and/or TTI, generate a clear welfare trade‐off by mitigating health costs and adversely affecting the economic activity.^3^ On the distributive side, the two types of policies provide health benefits to both susceptible and infected individuals, while also causing an economic and societal burden by reducing capital accumulation and consumption. If marginal net benefits resulting from the redistribution of resources through the TTI policy and/or through social distancing are positive (resp., negative), then it is convenient to strengthen (resp., relax) interventions to increase welfare. Social welfare is maximal only when the marginal benefits of interventions equal the corresponding marginal costs.

The social welfare function in (28) is consistent with previous works on the economic effects of COVID‐19, such as Acemoglu et al. (2020) and Alvarez et al. (2021), who made their objective function dependent on both COVID‐related economic and human losses to identify the optimal intensity of mitigation policies. Unlike them, however, we do not analyze the first‐best optimum but rather quantify the welfare effects associated with different policy measures (either alone or mixed) to assess which one might be the most desirable amongst those considered. In particular, our analysis aims at evaluating the welfare effects of specific policy rules represented by *constant intensities* of social distancing (measured by the proportion of people still at work, q) and TTI policies (whose intensity is tuned by the income tax rate, τ). Formally, the optimal problem proposed here is therefore constrained to the space of piecewise‐constant intervention function over the desired horizon.

This parsimonious approach is nonetheless rational compared with the myopic, adaptive piecewise‐constant programs adopted in most European countries during the second wave, which were adaptively following the changing epidemic conditions. From this viewpoint, our approach is in line with Atkinson and Stiglitz's (1980) second‐best optimality. The analysis that follows attempts to find the related constrained feasible optimal policies by assuming that the planner lacks the required (epidemiological) information to design a first‐best trajectory.

We now define $u\left(c\right)=\frac{c^{1-\sigma_{1}}}{1-\sigma_{1}}$ and $v\left(d\right)=\frac{d^{1+\sigma_{2}}}{1+\sigma_{2}}$, where $\sigma_{1},\sigma_{2}>0$ $\left(\sigma_{1}\neq 1\right)$ measure the constant elasticity of marginal utility of consumption and the constant elasticity of marginal disutility of COVID‐related mortality, respectively. The benevolent social planner maximizes (28) by choosing q and τ (separately or jointly) under (8) and taking the locking/unlocking thresholds as given. The numerical simulations are based on parameters in Tables 1 and 2 with the further parametric values: $\sigma_{1}=2$ (in line with the recent pre‐COVID‐19 empirical evidence, e.g., Havranek et al., 2015), $\sigma_{2}=1$ (Eichenbaum et al., 2020), ρ=0.04∕365 (on a daily basis). The values of W are scaled by a factor $1∕N_{0}$, where $N_{0}=\text{25,000,000}$ is the initial (pre‐COVID‐19) working population. The main results are presented in Section 5.1, which studies the case ϕ=0 and ε=1, and Section 5.2, which assumes individual noncompliance to health regulations. The welfare effects of (1) joint control programs (showing the possibility of *lockdown avoidance*), and (2) different combinations of ϕ and ε are postponed to the online appendix (Supporting Information).

### 5.1 Welfare analysis under ϕ=0,ε=1: Separate control programs

Results about separate control programs (i.e., social distancing/lockdown or TTI only) reveal—ceteris paribus—an inverted U‐shaped relationship between social welfare and either q or τ, showing the existence of social welfare maximizing lockdown intensity $q=q_{\max}$ (when no TTI is in place, i.e., τ=0) and social welfare maximizing tax rate $\tau =\tau_{\max}$ (when no lockdown is in place, i.e., q=1). This outcome is qualitatively robust to all choices of economic, demographic, and epidemiological parameters. However, from a quantitative standpoint, results show a dramatic dependence on the relative weight ω of the disutility of mortality compared with the utility of consumption. Additionally, the role of the discount rate ρ acquires importance over long time horizons, as expected.

**Figure 6:**
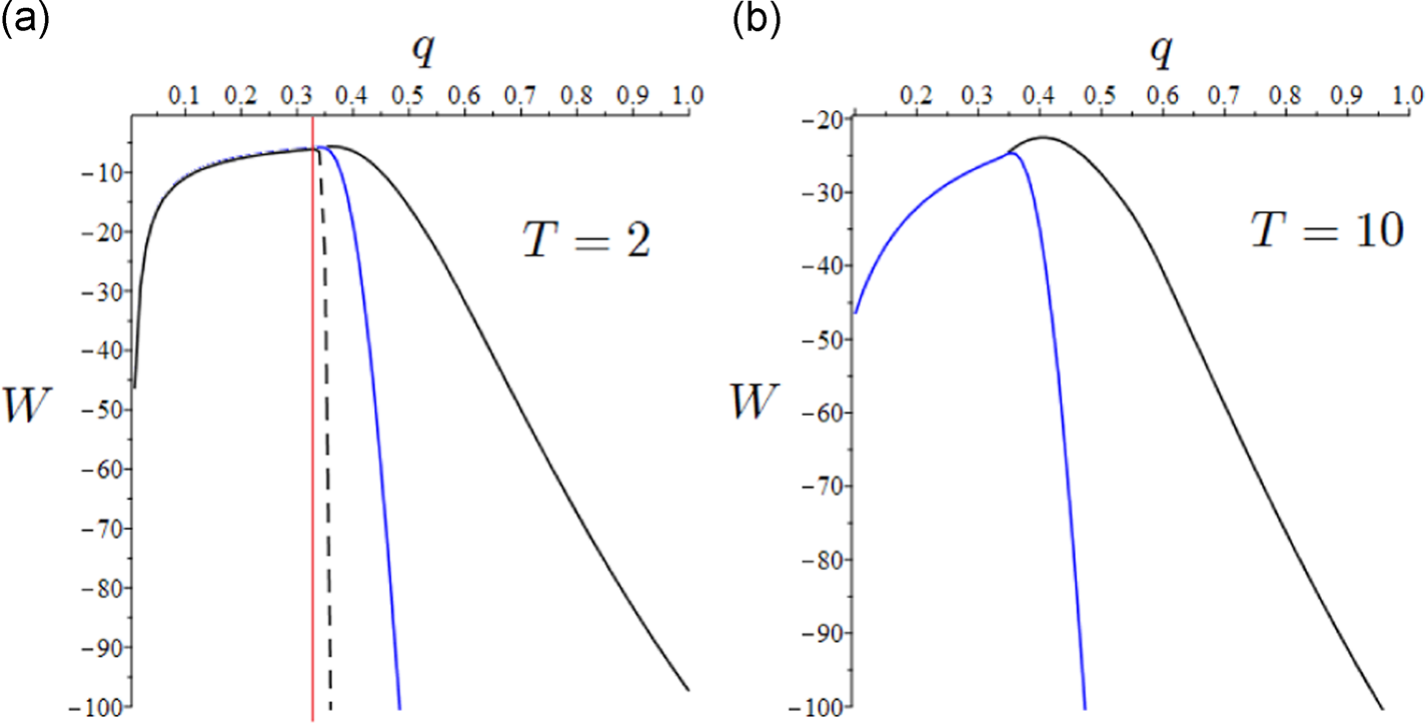
Optimal social distancing (for τ=0). Pattern of W as a function of q. (a) (T=2 years) considers three scenarios: $\omega =\omega_{\mathrm{low}}$ (black line), $\omega =\omega_{\mathrm{medium}}$ (blue line), and $\omega =\omega_{\mathrm{high}}$ (black dashed line). Optimal values: (i) $q_{\max}=0.36$ for $\omega =\omega_{\mathrm{low}}$; (ii) $q_{\max}=0.34$ for $\omega =\omega_{\mathrm{medium}}$; (iii) $q_{\max}=0.335$ for $\omega =\omega_{\mathrm{high}}$. The vertical red line is the value of q corresponding ${ℛ}_{0}=1$. (b) (T=10 years) considers two scenarios: $\omega =\omega_{\mathrm{low}}$ (black line) and $\omega =\omega_{\mathrm{high}}$. Optimal values: (i) $q_{\max}=0.41$ for $\omega =\omega_{\mathrm{low}}$; (ii) $q_{\max}=0.35$ for $\omega =\omega_{\mathrm{high}}$. The graphs of the curves overlap for small values of q

Figure 6 (in particular, Panel a) depicts social welfare as a function of q for distinct values $\omega_{\mathrm{low}}<\omega_{\mathrm{medium}}<\omega_{\mathrm{high}}$ of the relative weight ω mirroring different societal paradigms in terms of valuing direct epidemic versus economic costs (mutatis mutandis, comments to the other figures will follow straightforwardly), where $\omega_{\mathrm{low}}=1∕10^{7},\omega_{\mathrm{medium}}=10\cdot \omega_{\mathrm{low}}$, and $\omega_{\mathrm{high}}=10^{3}\cdot \omega_{\mathrm{low}}$. Figure 6a considers a short‐term horizon (T=2 years). This is perhaps longer than the COVID‐19 scenarios considered so far but it is consistent with the current evidence that Western countries will face the COVID‐19 threat for several further years despite vaccination. Figure 6a also reports a vertical line drawn at the value $q=q^{*}=0.333$ allowing ${ℛ}_{L}$ being brought nearby the unit threshold value (${ℛ}_{L}=1$), below which the government achieves epidemic control. On the one hand, for $\omega =\omega_{\mathrm{low}}$ (i.e., a society attributing a relatively low weight to human life than consumption, in terms of utility evaluation), the (constrained) optimal welfare, $W\left(q_{\max}\right)$, is obtained at $q=q_{\max}=0.36$, which does not prevent infection circulation. On the other hand, for $\omega =\omega_{\mathrm{high}}$ (i.e., a society attributing a relatively high weight to human life), the (constrained) optimal welfare is obtained at $q=q_{\max}=0.335$, which is just little above the epidemic persistence threshold. In this case, the welfare function dramatically drops for values larger than the optimum due to exceedingly large societal costs arising from COVID‐19 faster circulation. Intermediated results are obtained for the intermediate value of ω. *The almost catastrophic pattern of* W *for* $\omega =\omega_{\mathrm{high}}$ *represents a critical behavior suggesting the need for finely designed interventions aimed at pushing the lockdown intensity towards levels of transmission ensuring epidemic control (i.e*., ${ℛ}_{L}=1$ *or slightly lower, not much lower!), avoiding at the same time the unnecessary disruption of the economy*. From the latter standpoint, the vertical line at $q=q^{*}$ possibly represents the true societal optimum for a devastating epidemic as SARS‐CoV‐2 or any other socially transmitted infection with pandemic potential. To sum up, as far as ω increases the planner will be willing to apply more and more strict lockdown intensities at the optimum.

Figure 6b instead considers a longer‐term horizon (T=10 years) relying on the assumption that current epidemiological conditions are not modified by external circumstances, such as the advent of a vaccine (as argued in Section 1). It is easy to note that the main qualitative outcomes of Figure 6a are preserved but important quantitative differences appear. In particular, to avoid the harmful economic and societal consequences of the long‐term persistence of COVID‐19, the society—under the second‐best maximization—is willing to accept lower degrees of disease control. This is evident from the case $\omega =\omega_{\mathrm{low}}$ yielding a much larger optimal q (compared with Panel a), which now results at $q=q_{\max}=0.41$ instead of 0.36.^4^

**Figure 7:**
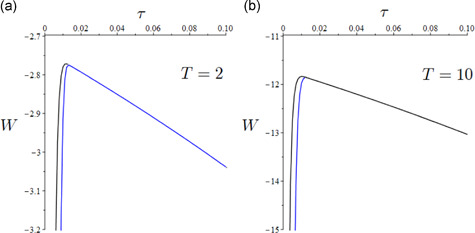
Optimal TTI (for q=1) pattern of W as a function of τ. (a) (T=2 years) optimal values: $\tau_{\max}=0.012$ for $\omega =\omega_{\mathrm{low}}$ (black line), and $\tau_{\max}=0.013$ for $\omega =\omega_{\mathrm{high}}$ (blue line). (b) (T=10 years) optimal values: $\tau_{\max}=0.010$ for $\omega =\omega_{\mathrm{low}}$ (black line), and $\tau_{\max}=0.012$ for $\omega =\omega_{\mathrm{high}}$ (blue line). The graphs of the two curves overlap for larger values of τ

Figure 7 shows, mutatis mutandis, the existence of a well‐defined (constrained) welfare maximizing TTI policy capable to keep the epidemic under control by tuning the tax rate τ. We observe that when $\omega =\omega_{\mathrm{high}}$ the society is willing to collect a larger tax burden than when $\omega =\omega_{\mathrm{low}}$ at the optimum. This implies a reduction in social welfare due to the larger cost of the intervention. *It is important to note that, ceteris paribus regarding the effectiveness in epidemic control, the TTI program yields higher social welfare than social distancing, as its expansion allows a larger reduction in both prevalence and COVID‐related mortality and a lower economic cost*. Last, we remark that the optimal tax rate increases with ω. This is because a society more inclined to protect human life might be willing to accept a higher tax burden for epidemic mitigation. These results support TTI as a mitigation policy, as is discussed in the concluding remarks.

### 5.2 Normative implications of noncompliance

The welfare analysis presented so far has focused on the pure effects of government interventions by disregarding the possible behavioral responses of individuals to mitigation measures. An obvious point is that an individual unanimously positive response to social distancing (and TTI) should not be taken for granted so that deterioration of control conditions might be expected due to, for example, resistant behavior. In this regard, some empirical evidence and speculations about noncompliance to social distancing during the first wave in Italy are reported in Briscese et al. (2020) and Carlucci et al. (2020).

This section presents a simple theoretical approach to behavioral responses within our Solow‐type setup. In doing so, it assumes that parameter q, which in the baseline scenario was the (chosen and realized) share of “fundamental” workers mandatorily compelled to work under social distancing, now represents the chosen share of fundamental workers, which however does not need to be realized one due to individual noncompliance (free‐riding). Therefore, the actual fraction at work during lockdown phases under partial noncompliance can be represented as $q+\lambda_{q}\left(1-q\right)$, where 1−q is the desired share of workers confined at home and $\lambda_{q}\in \left[0,1\right]$ the proportion of noncompliant workers. Likewise, noncompliance might also arise about TTI policies, for example, due to partial adhesion of individuals to testing/isolation programs. We represent this phenomenon by modifying the expression in (26) as follows: $\theta \left(g,i\right)=\frac{1-\lambda_{\tau }}{H+i}\cdot \frac{2\gamma g}{B+\eta g}$, where $\lambda_{\tau }\in \left[0,1\right]$ is the proportion of infected individuals who do not complain about the TTI program. Both these parameters can be widely heterogeneous amongst different communities depending on, for example, social norms, culture, social capital, education, politics, and so on.

**Figure 8:**
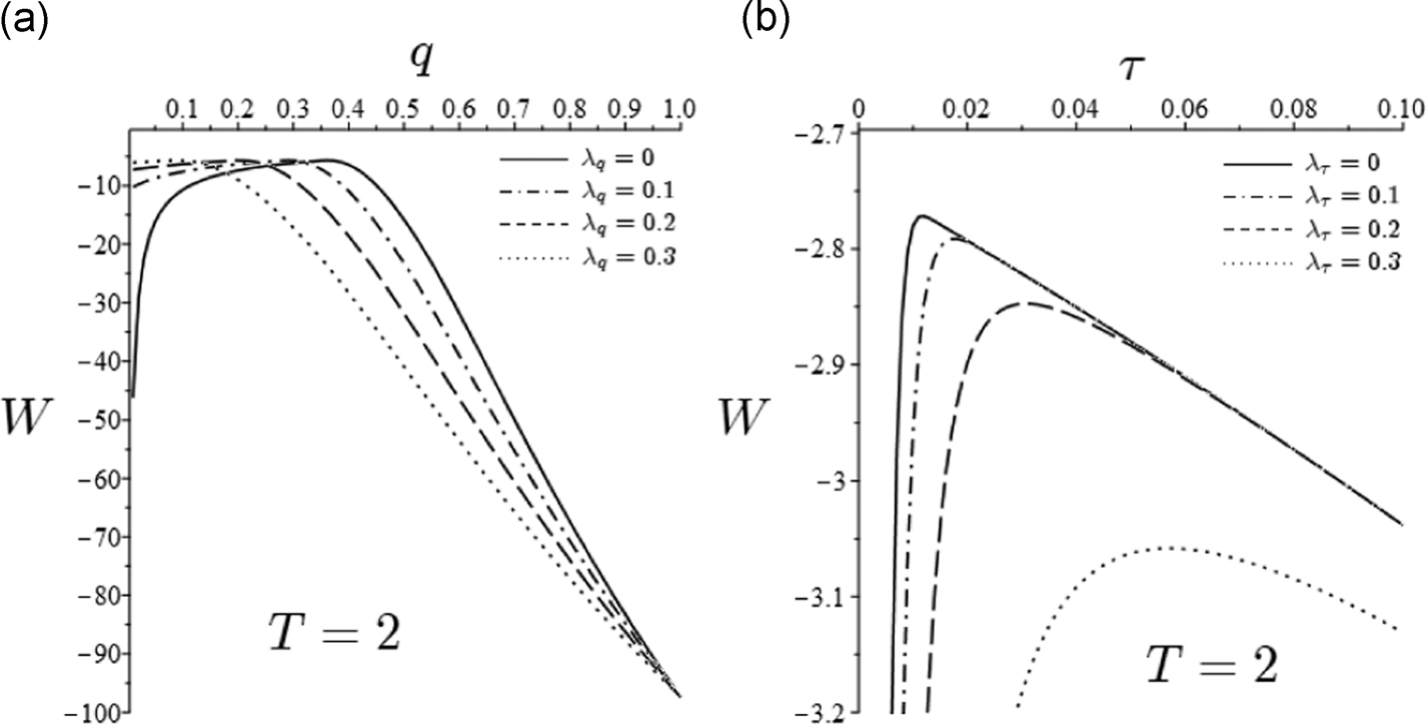
Case ϕ=0 and ε=1. (a) Patterns of W(q) for different values of $\lambda_{q}$ ($\omega =\omega_{\mathrm{low}}$ as in Figure 6a) and (b) patterns of W(τ) for different values of $\lambda_{\tau }$ ($\omega =\omega_{\mathrm{low}}$ as in Figure 6b)

The related results are reported in Figure 8. In particular, Panel (a) shows the social welfare effects of free‐riding in social distancing for increasing values of $\lambda_{q}$ (the scenario $\lambda_{q}=0$ was proposed in Figure 6a). In this regard, the second‐best social optimum value of q shifts leftward for increasing $\lambda_{q}$. This is expected as lower compliance forces the government to impose strengthened measures to maintain the desired mitigation target. The interpretation is simply that free‐riding increases the proportion of active workers, thereby improving production and capital accumulation but worsening epidemic control. Note that the maximum welfare remains unaltered (Figure 8a) exactly as it does the corresponding second‐best level of $R_{L}$. Note further that at lower compliance rates than those reported in Figure 8a, a threshold in $\lambda_{q}$ appears corresponding to which the government will be forced to declare *total nominal lockdown* (q=0) to maintain the mitigation target.

Unlike Figure 8a, the effects of free‐riding in the TTI policy (Figure 8b) show a monotonic worsening for increasing $\lambda_{\tau }$ simply because noncompliant isolation behaviors imply a subsequent increase in prevalence and a deterioration of control conditions. Other things being equal, this forces the government to increase taxation thereby reducing capital accumulation and production (and therefore social welfare).

Our theoretical analysis suggests that a higher degree of noncompliance requires introducing more stringent mitigation policies, either in the form of lockdowns or TTI. Therefore, knowing the extent to which individuals will follow public regulation is essential to implement effective policy measures. However, from an empirical perspective, calibration estimates of the degree of noncompliance in Italy are limited (Briscese et al., 2020; Carlucci et al., 2020). Briscese et al. (2020) document noncompliance to social distancing during the first epidemic wave, showing that compliance was affected by individual expectations about the length of social isolation measures: the longer the length of the lockdown compared with expectations, the more individuals will be unwilling to comply. Carlucci et al. (2020) further analyze the determinants of noncompliance to social distancing measures, showing that on average the degree of noncompliance was about 25%, but it was also characterized by high heterogeneity between different population groups. For our analysis, this suggests $\lambda_{q}≅0.25$, so that optimality would require the lockdown to be particularly severe, forcing about 90% of the workforce to remain idle to allow effective epidemic containment. Indeed, too high values of noncompliance would imply high economic costs to maintain the mitigation target that, in turn, would not allow overcoming the corresponding epidemiological costs.

## 6 DISCUSSION AND CONCLUDING REMARKS

The dramatic impact of the ongoing COVID‐19 pandemic on worldwide health and society, along with the current uncertainty due to the possible appearance of new virus strains, requires a careful understanding of the effectiveness of different intervention tools to control the spread of infectious diseases. We contributed to this issue by investigating the short‐, medium‐, and long‐term effects of a highly transmissible and fatal infection within a Solow‐type economic growth framework. Consistently, we analyzed the effects of the main nonpharmaceutical interventions (i.e., social distancing and TTI) employed during the pandemic, both from positive and normative perspectives.

First, we analyzed the long‐term interplay amongst an uncontrolled fatal infection with endemic potential, its demographic effects (on mortality and the population growth rate), and the steady‐state stock of capital, pinpointing the so‐called “world of opportunity after the plague” scenario (Gori et al., 2020; Young, 2005).

Second, the positive analysis showed that (i) resorting to a long‐term locking/unlocking policy may force the disease to stabilize at an artificial endemic state with a large susceptible proportion and a low degree of immunity, in turn suggesting that persistent lockdown strategies may not be a viable option, especially because they generate harmful consequences on capital accumulation, and (ii) publicly funded TTI measures have the potential to avoid future lockdowns by preventing future outbreaks. From this viewpoint, their current accumulation costs are a high‐return investment to be undertaken before the development of an effective vaccine.

Third, the normative analysis allowed us to determine the optimal lockdown intensity and the optimal tax rate to finance the TTI measures in a second‐best setting, highlighting some critical issues in the current debate on the COVID‐19 mitigation programs. There is a natural target of the lockdown intensity, which is rapidly bringing the epidemic reproduction (${ℛ}_{t}$) at—or just below—the critical threshold, thereby ensuring epidemic control and avoiding the disruption of the economy that necessarily arises when the transmission is brought to strongly subcritical levels (i.e., ${ℛ}_{t}<<1$). A massive continued TTI activity might obtain the same control result as the lockdown ensuring higher social welfare by avoiding the generalized closure of the economic activity. These results are at odds with the typical public health view (also prevailing during the COVID‐19 pandemic) which considers TTI as a pure containment strategy in an early epidemic phase and social distancing as the necessary mitigation strategy in more advanced phases. However, in a dramatic emergency period, like the one for COVID‐19, not dissimilar from a war condition, a national state might conceive a global emergency response based on a massive collection of taxes to be devoted to corresponding massive empowerment of the prevention system, including any available resource as, for example, digital tracing. This might be implemented by either transferring workers and related equipment, to public health or by, in a Keynesian spirit, hiring and retraining involuntary unemployed people. This should have been the clue—exploiting the low epidemic activity during summer 2020—to avoid the dramatic effects of the second pandemic wave in Western countries.

The article developed the simplest setting to analyze the trade‐off between the COVID‐19 health impact and the protection of economic activity, and as such it necessarily has some limitations. First, we do not deep the role of individual responses to changing epidemiological and economic conditions besides the simple analysis of noncompliance. For example, raising labor income taxation to finance a stricter TTI policy—as proposed in this study—may induce households to lower their labor supply thereby reducing the tax base, production capabilities, and the availability of resources to finance preventive health measures (Eichenbaum et al., 2020). Likewise, the increase in infection prevalence may force individuals to adopt protecting behaviors to reduce the risk of exposure, for example, by avoiding unnecessary mobility (Philipson, 2000). Second, in a first‐best optimal control scenario policymakers may design finely tuned interventions attempting to continuously respond to any change in epidemiological conditions. Third, one might include more details on the epidemiology of COVID‐19 (e.g., the uncertain duration of natural immunity, the role of age‐dependencies, the presence of asymptomatic carriers of infection, and so on; Kissler et al., 2020; Walker et al., 2020).

## CONFLICT OF INTERESTS

The authors declare that there are no conflict of interests.

## Supporting information

Supplementary Information

## Footnotes

1 Only a few countries worldwide, as China, were able to achieve epidemic suppression in the short term.

2 We recall that SARS‐CoV‐2 is an infection causing a large proportion of asymptomatic or paucisymptomatic cases. Testing/tracing activities might in general involve any category of individuals (susceptible, infected, and removed), but only the infected can be removed through testing and subsequent diagnosis and isolation.

3 The disease‐related mortality affects social welfare twofold: through the instantaneous utility function and the population size. The former captures the intratemporal effect of the epidemic (i.e., its short‐term impact on current generations), the latter its intertemporal effect (i.e., the long‐term impact also affecting future generations).

4 The locking/unlocking oscillations in Figure 3 are smoothed away by the integration in (28). This is because (i) such oscillations are regular due to the constant locking/unlocking thresholds and short lasting (about 50 days); (ii) we considered a minimum time horizon T=2 years (a fortiori, this holds for T=10).
