# Supplementary material for: COVID‐19 epidemic and mitigation policies: Positive and normative analyses in a neoclassical growth model
Source: J Public Econ Theory. 2021 Oct 21:10.1111/jpet.12549. Online ahead of print. doi: 10.1111/jpet.12549 (PMC8661658; doi:10.1111/jpet.12549)
Supplement: Supplementary file 1 — Supplementary Information [file JPET-9999-0-s001.pdf]

# COVID-19 epidemic and mitigation policies: positive and normative analyses in a neoclassical growth model

**Luca Gori*• Piero Manfredi†• Simone Marsiglio‡• Mauro Sodini§**

October 4, 2021

**Abstract**

The COVID-19 pandemic is still ravaging the planet, but its (short-, medium- and long-term) diverse effects on health, economy and society are far from being understood. This article investigates the potential impact of a deadly epidemic and its main non-pharmaceutical control interventions (social distancing versus testing-tracing-isolation, TTI) on capital accumulation and economic development at different time scales. This is done by integrating an epidemiological SIR model with a Solow-type growth model including public expenditure, as a parsimonious setting to offer insights on the trade-off between protecting human lives and the economy and society. The work clarifies (i) the long-term interactions amongst a deadly infection, demography and capital accumulation, (ii) the lack of viability of persistent social distancing measures, through an analytical characterisation, and the threat of policy-enhanced COVID-19 endemicity, (iii) the potentially high return on investments in TTI activities to avoid future lockdowns and related capital disruption. It also quantifies the welfare effects of a range of policies, confirming a counterintuitive role for tax-funded preventive investments aimed at strengthening TTI as more desirable interventions than generalised lockdowns.

**Keywords** Economic growth; Infectious diseases; COVID-19 transmission dynamics and interventions; Lockdown and testing; Welfare effects

**JEL Classification** C61; C62; J1, J22; O41; O47

---

*L. Gori (corresponding author), Department of Law, University of Pisa, Via Collegio Ricci, 10, I–56126 Pisa (PI), Italy, e-mail: luca.gori@unipi.it or dr.luca.gori@gmail.com., tel.: +39 050 22 12 847.

†P. Manfredi, Department of Economics and Management, University of Pisa, Via Cosimo Ridolfi, 10, I–56124 Pisa (PI), Italy, e-mail: piero.manfredi@unipi.it, tel.: +39 050 22 16 363, fax: +39 050 22 10 603.

‡S. Marsiglio, Department of Economics and Management, University of Pisa, Via Cosimo Ridolfi, 10, I–56124 Pisa (PI), Italy, e-mail: simone.marsiglio@unipi.it, tel.: +39 050 22 16 382, fax: +39 050 22 10 603.

§M. Sodini, Department of Law, University of Naples ‘Federico II’, Via Mezzocannone 16, I–80134 Naples (NA), Italy, e-mail: mauro.sodini@unina.it. Department of Finance, Faculty of Economics, Technical University of Ostrava, Ostrava, Czech Republic.

# Online appendix

This appendix aims at discussing some mathematical details of the economic-epidemiological study presented in the main text by also offering different perspectives about the second-best optimal policies against COVID-19. In doing so, it considers the normative analysis of 1) joint control programmes under the assumption under the baseline scenario discussed in the main text, i.e., $\phi = 0$ and $\varepsilon = 1$, and 2) the possible combinations of the mortality indicator, $\phi = \{0, 1\}$, with the welfare criterion, ranging from Benthamite ($\varepsilon = 1$) to Millian ($\varepsilon = 0$) utilitarianism.

# Proof of Proposition 2

The dynamics of the SIR subsystem in (8) presented in the main text is independent of the evolution of $k$. About the stability of the subsystem given by the epidemiological equations in (8) we refer to Theorem 2.1 in Busenberg and van den Driessche (1990). Regarding the fourth equation, we have that when the SIR subsystem respectively converges to the infection-free equilibrium or the endemic equilibrium, the dynamics of $k$ are asymptotically governed by $\dot{k} = xAk^{\alpha}\ell^{1-\alpha} - (b - \mu - \hat{\imath}\mu_c + \delta)k$, where $\hat{\imath} = 0$ and $\ell = 1$, or $\hat{\imath} = i^*$ and $\ell = 1 - i^*$, respectively. The result of the proposition follows immediately as in both cases the last equation of the system is Bernoulli-like.

# Normative analysis under $\phi = 0$, $\varepsilon = 1$: joint programmes (lockdown avoidance)

The normative analysis presented in the main text has concentrated on the existence of possible (second-best) optimal values of 1) the parameter govering the extent of social distancing ($q$) in the absence of TTI ($\tau = 0$), and 2) the tax rate governing the extent of TTI ($\tau$) in the absence of social distancing ($q = 1$). However, the social distancing and TTI programmes can be combined with the aim at maximising the social welfare function (28) in the main text subject to (8) and taking the locking/unlocking thresholds as given. In this case, results show that at low or moderate TTI efficiency (as measured by $\eta$) an internal solution for $q$ exists, meaning that a certain degree of social distancing is anyhow necessary in that case. However, for a sufficiently high TTI efficiency, there is always an optimal tax rate ($\tau = \tau_{\max}$) allowing *lockdown avoidance* ($q = q_{\max} = 1$) in both the short ($T = 2$) and longer terms ($T = 10$). With our parameter choices, this holds for any $\omega > 0$ and the welfare-maximising tax rate is larger the larger $\omega$, as the society is willing to accept a higher tax burden to grasp the benefits of reducing COVID-related mortality. Lockdown avoidance may follow two distinct trajectories (Table A1). In the short term ($T = 2$) when $\omega = \omega_{low}$ the amount of resources collected through (the welfare-maximising) taxation is not sufficient to avoid infection prevalence exceeding the locking threshold ($T_L$) at some stages of the epidemic (see Fig. 5 in the main text for comparison purposes). However, the reduction in epidemiological costs and the corresponding increase in economic benefits are larger than those related to the lockdown policy, which is therefore not implemented. When $\omega = \omega_{high}$, the resources collected through taxation at the optimum are high enough to almost always avoid prevalence to exceed $T_L$, though maximal welfare is lower than when

$\omega = \omega_{low}$ due to the larger amount of resources the society is willing to collect in that case. As for long-term horizons ($T = 10$), results are similar to short-term ones, so that the planner chooses not to declare lockdown in either case $\omega_{low}$ and $\omega_{high}$ though in some phases infection prevalence exceeds the $T_L$ threshold. However, the TTI-related expenditure decreases over time due to the reduced number of infections compared to the short term causing a reduction in epidemiological costs and an increase in economic benefits.

| $T$ | $\omega$ | $q_{\max}$ | $\tau_{\max}$ | $W(q_{\max}, \tau_{\max})$ |
|---|---|---|---|---|
| 2 | $\omega_{low}$ | 1 | 0.012 | $-2.7716$ |
| 2 | $\omega_{high}$ | 1 | 0.013 | $-2.7750$ |
| 10 | $\omega_{low}$ | 1 | 0.010 | $-11.8343$ |
| 10 | $\omega_{high}$ | 1 | 0.012 | $-11.8567$ |

**Table A1**. Welfare analysis of joint intervention programmes: $\omega_{low} = 1/10^7$ and $\omega_{high} = 10 \cdot \omega_{low}$.

## Normative analysis: comparison of different choices on parameters $\phi$ and $\varepsilon$

This section offers a broader perspective about the second-best optimal policies against COVID-19 analysed in the main text (Section 5) by considering the possible combinations of the mortality indicator, $\phi = \{0, 1\}$, with the welfare criterion, ranging from Benthamite ($\varepsilon = 1$) to Millian ($\varepsilon = 0$) utilitarianism. Results show (compare Figs. A1 and A2 with Figs. 6 and 7 in the main text) marked robustness for both dimensions. In particular, social welfare still exhibits a hump-shaped pattern with $q$ and $\tau$ regardless of the choice on the pair $(\phi, \varepsilon)$. Qualitatively, this result was not unexpected as COVID-19 mortality has been tuned to crudely mirror real-world mortality in the working population, implying negligible effects on the size of the general population $N$, which follows an almost constant trend. This implies that the COVID-related welfare effects are not significantly affected by the adopted utilitarian criterion. We pinpoint that the relative weight of the disutility of COVID-19 mortality ($\omega > 0$) in Figs. A1 and A2 has been set to obtain the same levels of the second-best optimal values of the control intervention parameters $q$ and $\tau$.

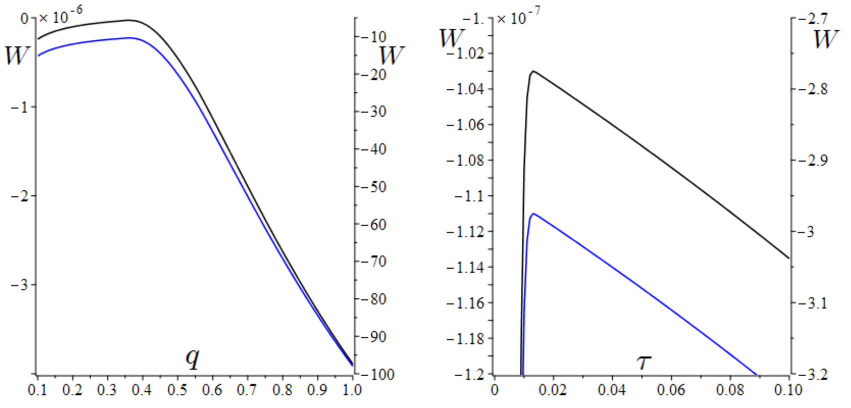


(a) (b)

**Figure A1**. Case $\phi = 0$. Panel (a): comparison between the patterns of $W(q)$ for $\varepsilon = 0$, $\omega = 6.4 \cdot 10^6$ (blue curve, left axis) and $\varepsilon = 1$, $\omega = 1/10^7$ (black curve, right axis). The optimal value is $q^* = 0.36$. Panel (b): comparison between the patterns of $W(\tau)$ for $\varepsilon = 0$, $\omega = 6.4 \cdot 10^6$ (blue curve, left axis) and $\varepsilon = 1$, $\omega = 1/10^7$ (black curve, right axis). The optimal value is $\tau^* = 0.013$.

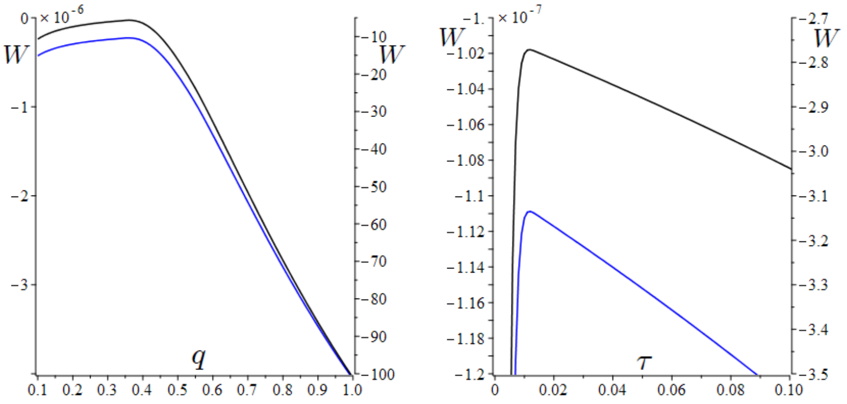


(a) (b)

**Figure A2**. Case $\phi = 1$. Panel (a): comparison between the patterns of $W(q)$ for $\varepsilon = 0$, $\omega = 6.4 \cdot 10^6$ (blue curve, left axis) and $\varepsilon = 1$, $\omega = 1/10^7$ (black curve, right axis). The optimal value is $q^* = 0.36$. Panel (b): comparison between the patterns of $W(\tau)$ for $\varepsilon = 0$, $\omega = 6.4 \cdot 10^6$ (blue curve, left axis), and $\varepsilon = 1$, $\omega = 1/10^7$ (black curve, right axis). The optimal value is $\tau^* = 0.012$.

# References


[1] Busenberg, S., van den Driessche, P., 1990. Analysis of a disease transmission model in a population with varying size. Journal of Mathematical Biology 28, 257–270.
